# Bacchus by Caravaggio as the Visual Diagnosis of Alcohol Use Disorder from the Fifth Edition of the Diagnostic and Statistical Manual of Mental Disorders (DSM-5)

**DOI:** 10.3389/fpsyt.2013.00086

**Published:** 2013-08-08

**Authors:** Carolina L. Haass-Koffler, George A. Kenna

**Affiliations:** ^1^Center for Alcohol and Addiction Studies, Department of Behavioral and Social Sciences, Brown UniversityProvidence, RI, USA; ^2^Bouvé College of Health Sciences, Northeastern UniversityBoston, MA, USA; ^3^Department of Psychiatry and Human Behavior, Brown UniversityProvidence, RI, USA

**Figure 1:**
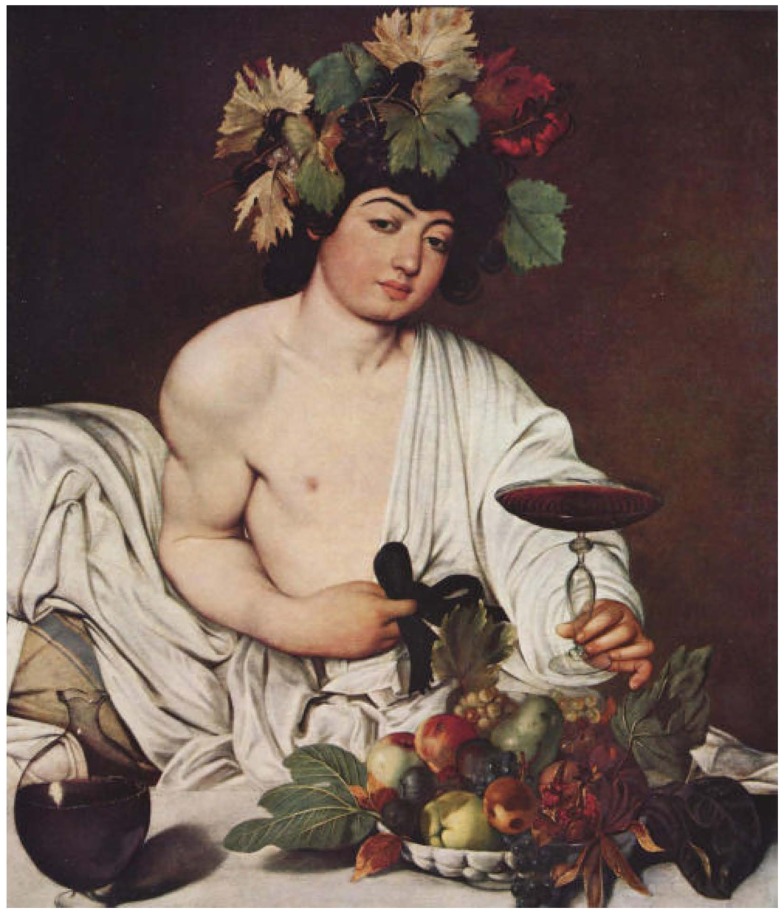
**Bacchus (1596), Michelangelo Merisi from Caravaggio**. Oil on canvas, 95 × 85 cm, *Galleria degli Uffizi*, Florence, Italy. Image reproduced with permission from the *Soprintendenza Speciale per il Patrimonio Storico Artistico e Etnoantropologico per il Polo Museale*, Florence, Italy.

For his Bacchus (Figure 1), Michelangelo Merisi from Caravaggio (1571–1610) did not choose a model that resembles the classical idolized God of wine represented in the past. The figure is not the Greek Dionysus, a strongly built, mature male wearing a robe. The figure is not the young Roman Bacchus represented with vine and dripping honey who seems to be free from self-consciousness. Instead, he chose a young boy without refined physical traits. He depicted a vulgar youngster, dressed as Bacchus, who put on a costume in an irreverent manner, waiting to drink the wine in the chalice, collecting the few *scudi* (Roman currency of the day) for modeling in order to run to the tavern at the end of the street for more drinking. Because of Caravaggio's lack of money for hiring models and his love for alcohol, many art historians suggested that the painting is a self-portrait. Furthermore, because the figure holds the chalice containing the red wine with his left hand, it has hypothesized that Caravaggio used the help of a mirror (1, 2).

The dangerously inclined chalice with rippling wine, combined with the wine carafe with a very small pedestal, makes for a very wobbly scene. The wine, a Eucharistic symbol, could spill from the chalice held by the model and splatter from the carafe, which contains a small reflection of himself. The entire image gives us the concept of time where, in a moment of instability, a precariously balanced image of life could spiral into chaos.

The basket of fruit, common trait in Caravaggio's paintings, is not just a decoration of beautiful foliage and succulent produce. The painting includes both a collection of savory and decayed fruits, framed by flourishing fresh green and drying yellow-brown leaves. The still life part of this painting embodies the simultaneous presence of health and sickness, life and death. This cycle of life however is not depicted only to represent the transient and inevitable nature of existence that would be depicted the following century in the *vanitas* by Flemish painters. The still life that shares the space with this Bacchus reminds the viewer of the futility of pleasure and the certainty of death, but lacks the symbols of hope and renewal against darkness, which are expressed by the presence of insects. Furthermore, the symbol of power, represented in fine arts by a crown, is in this figure illustrated by a crest of grapes and senescing leaves that reiterate the deterioration of life.

With his depiction of Bacchus, Caravaggio used photo-like realism to deliver an accurate and precise diagnosis of Alcohol Use Disorder (AUD). With the model's features, Caravaggio was able to depict the neurological and psychiatric symptoms of alcoholism. The skin rosacea and dirty fingernails allude to nutritional deficiency and poor personal hygiene. He showed both the impaired fine and gross motor movements; the hand tremor resulted in the undulating wine inside the chalice, and the possibility exists of losing the balance of the model's right elbow and hitting the fragile carafe. The dilated blood vessels showed by the flushed face, swollen eyelids, and red hands, are the physical characteristics of an individual suffering from AUD. In his direct realism, Caravaggio also chose to depict himself in another self-portrait during an illness characterized by strong jaundice (Sick Bacchus, 1594). Caravaggio, probably being an alcoholic himself, was spending a great deal of time in taverns. He had seen chronic alcoholic individuals with jaundice and had witnessed their death (assumed to be from liver failure due to cirrhosis) (3).

Using the symbolism encoded in the still life, Caravaggio was able to illustrate the psychological characteristics of alcoholic individuals. Caravaggio depicted the decreasing of inhibition and the increasing of seductive behaviors of the model by using eternal life symbolism. The pomegranate is cracked, inferring fall from grace and open to sinful nature. The white and black grapes crowning the head of the model, which signify lewdness and lustful thoughts, are ripened and pungent, while the leaves, symbolizing loss of innocence, are yellowing and drying. There are no butterflies or moths to evoke the idea of rebirth, but just a codling moth entrance hole. This portrait is a sad and gloomy face of life that does not comprise salvation and resurrection.

Many of the revisions in the Fifth Edition of the Diagnostic and Statistical Manual of Mental Disorders (DSM-5, dsm5.org) are intended to provide diagnosis and clinical care support to non-psychiatrists (since primary care physicians are most likely to be the first to assess psychiatric symptoms) (4). One of the major changes is the merger of the two diagnoses of Alcohol Abuse and Alcohol Dependence into a single diagnosis called Alcohol Use Disorder (AUD). The medical community has perceived the merger of the criteria positively since the DSM-IV term dependence was restricted only to patients experiencing withdrawal during treatment (5) leaving some patients “diagnostic orphans” (6). In the DSM-5, those patients are included in the AUD criterion and continue receiving medical treatment as in other psychiatric disorders (7).

The Bacchus, according to the DSM-IV criteria would not meet the full requirements for an Alcohol Dependence diagnosis because he is not an individual experiencing alcohol withdrawal during treatment and he does not appear to suffer of severe dependence (8). Furthermore, he probably would not meet the criteria of Alcohol Abuse since as a model, he is performing a job responsibility. Every aspect of the painting, however, suggests that Bacchus is suffering from AUD; the Bacchus would be a “diagnostic orphan.”

Caravaggio challenged society with his controversial and revolutionary art. He developed a realism that forced his contemporaries to see the actual face of alcoholism. That image depicted more than 400 years ago is today the pictorial representation of AUD and patients with the updated DSM-5 criteria will obtain a diagnosis and will receive treatment.
